# Structural and Functional Insights into the C-terminal Fragment of Insecticidal Vip3A Toxin of *Bacillus thuringiensis*

**DOI:** 10.3390/toxins12070438

**Published:** 2020-07-05

**Authors:** Kun Jiang, Yan Zhang, Zhe Chen, Dalei Wu, Jun Cai, Xiang Gao

**Affiliations:** 1 State Key Laboratory of Microbial Technology, Shandong University, Qingdao 266237, China; jiangkun@sdu.edu.cn (K.J.); yanzhang1991@sdu.edu.cn (Y.Z.); zhechen@mail.sdu.edu.cn (Z.C.); dlwu@sdu.edu.cn (D.W.); 2 Helmholtz International Lab, Shandong University, Qingdao 266237, China; 3 Department of Microbiology, College of Life Sciences, Nankai University, Tianjin 300071, China; caijun@nankai.edu.cn

**Keywords:** *Bacillus thuringiensis*, Vip3A, 3D-structure, mode of action, biological control

## Abstract

The vegetative insecticidal proteins (Vips) secreted by *Bacillus thuringiensis* are regarded as the new generation of insecticidal toxins because they have different insecticidal properties compared with commonly applied insecticidal crystal proteins (Cry toxins). Vip3A toxin, representing the vast majority of Vips, has been used commercially in transgenic crops and bio-insecticides. However, the lack of both structural information on Vip3A and a clear understanding of its insecticidal mechanism at the molecular level limits its further development and broader application. Here we present the first crystal structure of the C-terminal fragment of Vip3A toxin (Vip3Aa11_200–789_). Since all members of this insecticidal protein family are highly conserved, the structure of Vip3A provides unique insight into the general domain architecture and protein fold of the Vip3A family of insecticidal toxins. Our structural analysis reveals a four-domain organization, featuring a potential membrane insertion region, a receptor binding domain, and two potential glycan binding domains of Vip3A. In addition, cytotoxicity assays and insect bioassays show that the purified C-terminal fragment of Vip3Aa toxin alone have no insecticidal activity. Taken together, these findings provide insights into the mode of action of the Vip3A family of insecticidal toxins and will boost the development of Vip3A into more efficient bio-insecticides.

## 1. Introduction

The entomopathogenic bacteria *Bacillus thuringiensis* (Bt), is the most widely used microbial insecticide in the world [1,2]. It is renowned for its ability to produce insecticidal crystal proteins (Cry toxins) during its sporulation phase, which have been widely used in the prevention and control of agricultural pests through the development of transgenic plants or Bt-based biopesticides [3,4,5]. However, many pests are not sensitive to Cry toxins, and the development of insect resistance has also been reported [1,6,7]. The successful application of Cry proteins, coupled with their limitations, has spurred on intensive research seeking to identify and characterize novel classes of insecticidal toxins that can be developed for agricultural purposes.

Vegetative insecticidal proteins (Vips), which are produced by Bt during its vegetative stages, have a wide spectrum of insecticidal activity, especially against lepidopteran pests [8]. To date, ~150 distinct Vip toxins have been identified, which have been classified into four families (Vip1, Vip2, Vip3 and Vip4) based on their sequence similarity [9]. Among the Vip toxin family, Vip3A toxins are the most abundant and most studied [8]. Compared with known Cry toxins, Vip3A toxins share no sequence homology, bind to different receptors [10,11,12,13], and lack cross-resistance [14,15,16,17], therefore they are considered as ideal options to complement and expand the use of Bt in crop protection and resistance management. At present, the Vip3Aa toxin is the only family member that has been used in commercial transgenic crops together with Cry toxins, and no field-evolved resistance has yet been reported [1,8,18]. However, the lack of structural information and incomplete understanding of its mechanisms of action have severely limited the further development of Vip3A as a tool in pest control. 

Vip3A toxins are large proteins (~789 amino acids) consisting of a conserved N-terminus and a variable C-terminal region. The ~88kDa Vip3A protoxin could be digested by insect midgut juices into two fragments: a ~20 kDa fragment corresponding to the N-terminal 198 amino acids, and a ~65 kDa fragment corresponding to the C-terminal fragment of Vip3A protein, which is regarded as an essential step for its activation and toxicity [12,19,20,21,22,23]. Since their discovery in 1996 [24], Vip3A proteins have been the subject of intensive research. It has been reported that Vip3A stimulates membrane pore formation and apoptosis upon binding to target cells, which is proposed to be responsible for its cytotoxic effects [12,25,26,27]. The scavenger receptor class C like protein (Sf-SR-C) and the fibroblast growth factor receptor (Fgfr) have been reported as potential receptors for Vip3A [10,11]. Vip3Aa16 and Vip3Af1 have been subjected to in silico modelling, and three domains and five domains were proposed respectively [28,29]. Quan et al. propose a map of Vip3Af protein with five domains based on the altered protease digestion patterns through the Vip3Af alanine mutants [23]. In addition, Vip3Ag protoxin and the trypsin-activated toxin were found to be a potential tetrameric complex according to the surface topology obtained by transmission electron microscopy [20]. Recently, Zheng et al. reported the crystal structure of a Vip3B protoxin like protein: Vip3B2160 [30], which shares around 60% sequence identity to Vip3A. The overall structure of Vip3B2160 shows a five-domain organization and forms a novel tetramer structure assembly. However, the atomic structure of Vip3A is still not available, which makes it difficult to reveal the relationship between its structure and function accurately.

Here, we report the crystal structure of the C-terminal fragment of Vip3A toxin (Vip3Aa11_200–789_). The structure shows a four-domain organization which is likely to be conserved for all insecticidal Vip3A family toxins. We identify conserved hydrophobic α-helices in domain II, which we predict to be involved in the membrane insertion process. Structure-guided cell binding assays reveal that domain III may have a central role in host cell targeting and binding of Vip3A toxins. Structural analysis indicates that Vip3A toxins have potential for glycan binding through domains IV and V. Together, our structural and functional studies provide new insights into the molecular mechanisms underlying the mode of action of insecticidal Vip3A toxins.

## 2. Results

### 2.1. Overall Structure of Vip3Aa11_200–end_

We used a Vip3A toxin from Bt strain C9, which has been named Vip3Aa11 (GenBank accession No. AY489126.1) in this study. Full-length Vip3Aa11 consists of 789 amino acids, which have been demonstrated to be digested between residues K198 and D199 by insect midgut juice [19,20,21]. We initiated our crystallization trial with both Vip3Aa11 protoxin and Vip3Aa11_199–end_. Using spare matrix crystallization screening, we only identified one condition that yielded needle-shaped crystals of Vip3Aa11 protoxin. However, the crystals diffracted to only ~15 Å and could not be improved despite extensive effort. No crystals were observed for the Vip3Aa_199–end_ construct despite screening more than 1000 crystallization conditions. However, when we deleted the N-terminal amino acid (Asp199) from the Vip3Aa11_199–end_, we obtained the crystal of Vip3Aa11_200–end_, which diffracted to ~6 Å. Through the addition of an N-terminal MBP (Maltose Bind Protein) tag, we were able to isolate crystals with improved diffraction. The structure was solved using a combination of anomalous phasing with a selenomethionine derivative crystal of Vip3Aa_200–end_ and molecular replacement using MBP as a model in native crystals. The final structure of Vip3Aa11_200–end_ was refined to a 3.2 Å resolution with R and R_free_ values of 0.1980 and 0.2389, respectively (Table S1). 

**Figure 1:**
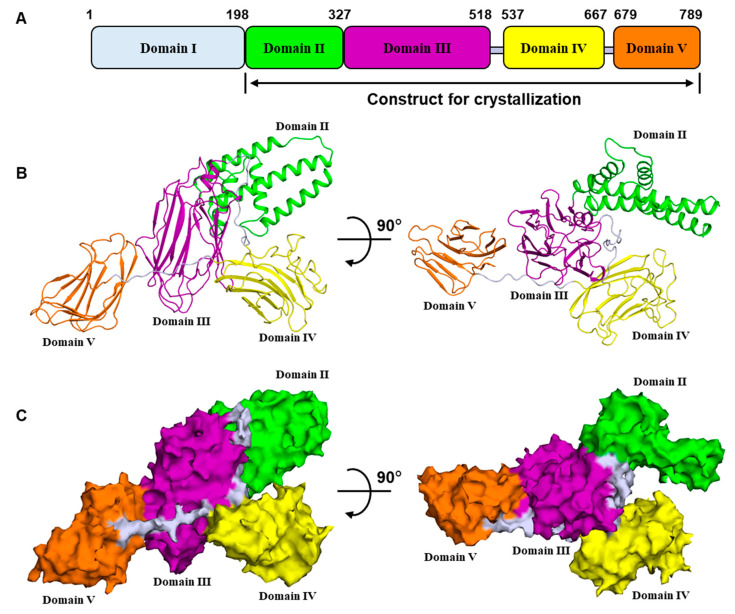
Overall structure of vegetative insecticidal protein Vip3Aa11_200–end_. (**A**) Domain organization of Vip3A. (**B**) Two views of the overall structure of Vip3Aa11_200–end_ monomer colored as in (**A**). (**C**) Two views of the surface model of Vip3Aa11_200–end_ monomer colored as in (**A**). The black arrow indicates the angle of rotation around the central axis.

The structure shows that the Vip3Aa11 C-terminal fragment is comprised of four domains (Figure 1A,B). Vip3Aa11 could be digested between residues K198 and D199, and residues 1–198 are lacking in our structure. For a better description based on the full-length Vip3A, we assume that Vip3Aa11_1–198_ is a separate domain. Then the protoxin can be divided into five domains, starting from N-terminus: domain I, 1–198; domain II, 199–327; domain III, 328–518; domain IV, 537–667; and domain V, 679–789 in Vip3Aa11 (Figure 1A and Figure S1). The overall structure of the Vip3Aa11_200–end_ resembles a lobster, wherein domains II and III form the body, and domains IV and V are the claws (Figure 1B,C). The connection between domain II and domain III is compact. However, domains III/IV and IV/V are connected by long and flexible loops, which indicates that the relative locations and orientations of these two domains could change under different biological circumstances. There are over 100 known proteins of the Vip3A family. Based on their high degree of sequence conservation and previous studies [8], they are very likely to share similar overall structures and domain compositions.

The crystal belongs to the P2_1_ space group and four MBP-Vip3Aa11 molecules were found in one asymmetric unit (Figure S2). These four molecules form two dimers in different orientations. PISA [31] determined that there was limited interaction between the two dimers, indicating that their association was caused by crystal packing. Notably, the two Vip3Aa11 molecules in the “dimer” showed moderate conformational variations, with a core root mean square deviation (r.m.s.d) of 1.234 Å among 468 Cα atoms (Figure S3). Superimposition of separate domains between the two molecules revealed better alignment for domains III, IV and V, but not for domain II (Figure S3), suggesting that domain II might potentially be involved in the conformational changes during the activation of Vip3A toxins. Due to their high similarity, we used the monomeric structure of Vip3Aa11_200–end_ for subsequent analysis.

### 2.2. Domain II Contains a Conserved Hydrophobic Architecture

**Figure 2:**
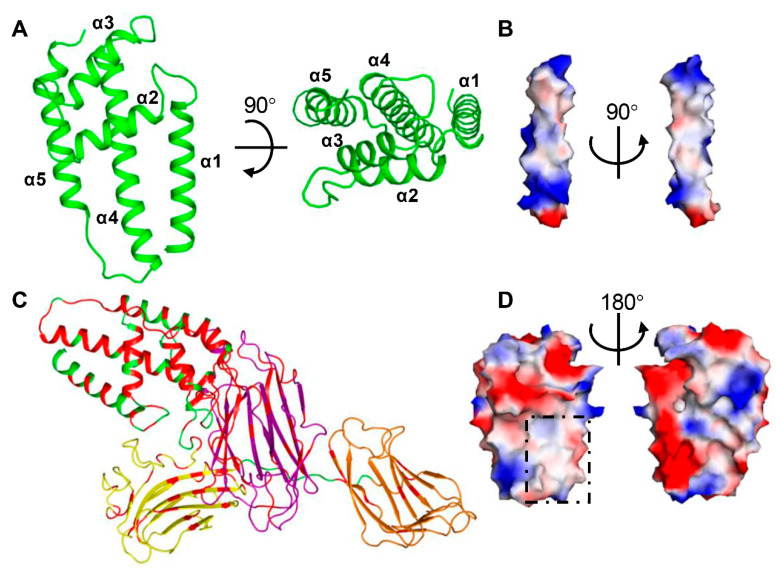
Domain II of Vip3Aa11 shows a conserved hydrophobic surface. (**A**) Two views of structure of Vip3Aa11 domain II shown as a ribbon cartoon. (**B**) Two views of the surface model of helix α4 from domain II show its surface charge distribution. (**C**) The highly conserved amino acid residues from Vip3 family sequence alignment (Figure S1) are highlighted in the Vip3Aa structure with red color. (**D**) Two views of the surface model of Vip3Aa11 domain II show its surface charge distribution. The conserved hydrophobic surface is highlighted by black square. (**B**,**D**) The surface is colored as the basis of electrostatic potential with positive charged surface in blue and negatively charged area in red. The black arrow indicates the angle of rotation around the central axis.

Domain II of Vip3Aa11 (residues199–327) consists of five helices, which form two layers (Figure 2A). The outer layer facing the solution contains two short helices, α2 and α3, while the inner layer that contacts with domain III consists of three anti-parallel helices α1, α4 and α5. The outer layer contacts with the upper portion of the inner layer and is almost perpendicular to the inner layer. Among these five helices, helix α4 is the longest. It spans around 45 Å and contains 30 amino acid residues, starting from E267 at the N-terminus to L296 at the C-terminus. Electrostatic surface potentials analysis shows that the majority of charged and polar amino acid residues locate at the N-terminal and C-terminal ends of helix α4 (Figure 2B). For the middle portion of helix α4, from F274 to L289, 75% amino acid residues are hydrophobic residues. Sequence alignment through Vip3 family shows that the hydrophobic region of helix α4 is very much conserved and it is also the most agminated hydrophobic region of Vip3 family proteins (Figure S1). Close to helix α4, helix α1 also shows several conserved hydrophobic amino acid residues facing helix α4 (Figures S1 and S4).

Based on the sequence alignment (Figure S1), all the conserved amino acid residues were highlighted on the Vip3Aa11_200–end_ structure (Figure 2C). This shows that the sequence of domain II is highly conserved (about 62%), only slightly lower than that of domain I (about 68%). Electrostatic surface potential analysis shows that there is an obvious hydrophobic surface, which is mainly contributed to by the conserved helix α1 and α4 (Figure 2D). 

### 2.3. Domain III Is Involved in Cell Binding of Vip3A Toxin

**Figure 3:**
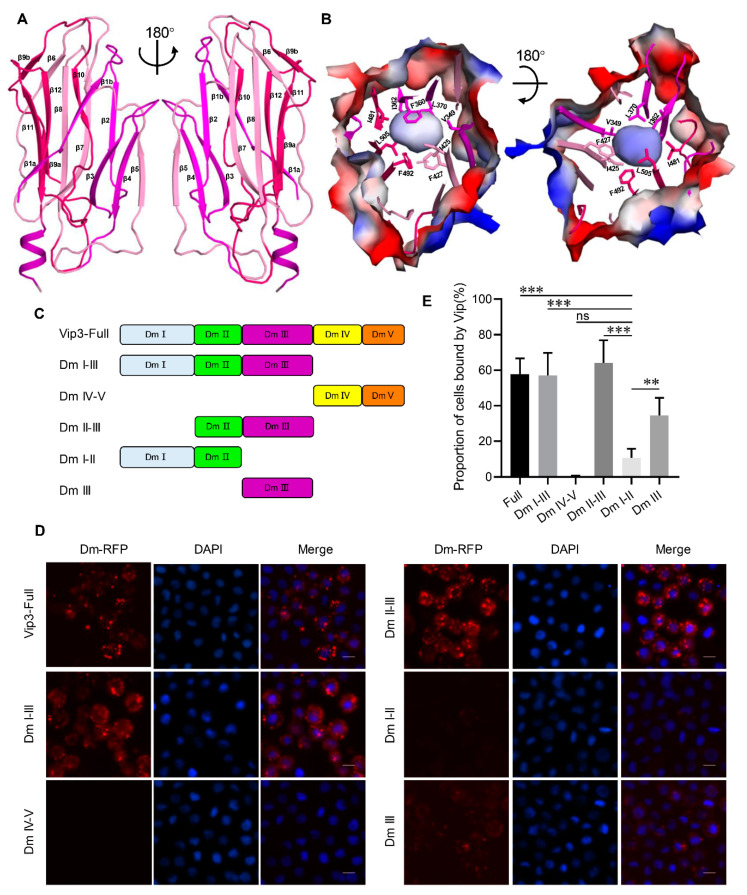
Domain III is a potential receptor binding domain. (**A**) Overall structure of Vip3Aa11 domain III shown as a ribbon cartoon. Two views of three antiparallel β sheets from domain III are shown in three different colors, the black arrow indicates the angle of rotation around the central axis. (**B**) Two views of the surface model of domain III of Vip3Aa11. Inside the domain III, there is a conserved hydrophobic core, and the conserved hydrophobic amino acid residues from three antiparallel β sheets are shown as sticks, the black arrow indicates the angle of rotation around the central axis. (**C**) The schematics of C-terminal RFP (red fluorescent protein)-tagged Vip3Aa and its truncation derivatives. (**D**) Fluorescence microscope images of Sf9 cells treated with Vip3Aa-RFP or its truncations, which were labeled with C-terminal RFP tag, for 6 h. Nuclei are stained with DAPI (blue). (**E**) Quantification of the number of Sf9 cells that can be bound by RFP-tagged Vip3Aa and its truncations of Figure 3D in a blind fashion (*n* = 100 cells per sample). Data are expressed as the mean ± SD from three independent experiments. ns, nonsignificant; **, *p* < 0.01; ***, *p* < 0.001. Scale bar: 10 μm.

Domain III of Vip3Aa11 (residues 328–518) consists of twelve β strands and one short α-helix at the C-terminal end (Figure 3A). Twelve β strands comprise three antiparallel β sheets sharing a similar “Greek-key” topology (Figure 3A) with a hydrophobic center featuring highly conserved residues V349, F360, I362 and L370 from β sheet I, I425 and F427 from β sheet II, and I481, F492 and L505 from β sheet III (Figure 3B). The results from the DALI server [32] showed that the fold of domain III is similar to that of domain II of the three domain Cry (3d-Cry) family of insecticidal toxins, which has been shown to be involved in host cell receptor recognition and binding [4]. We therefore sought to explore whether domain III serves as a receptor binding domain for Vip3A toxins.

To explore this hypothesis, we used *Spodoptera frugiperda* cells (Sf9 cells), which have previously been shown to be specifically targeted by Vip3 toxins [11,33]. To determine which domain(s) of Vip3Aa11 interact with Sf9 cells, we carried out fluorescence-based cell binding assays using different C-terminal RFP-tagged Vip3Aa truncation derivatives (shown schematically in Figure 3C). As shown in Figure 3D,E and Figure S5, while domain IV-V does not show detectable binding to Sf9 cells, the binding of a construct featuring only domain I-III or II-III to Sf9 cells is indistinguishable from that of full-length Vip3Aa. The interaction of domain III alone with Sf9 cells is significantly stronger than that of the domain I-II construct, indicating that domain III may have a central role in Vip3A receptor binding to Sf9 cells. In addition, Domain II-III shows higher binding than Domain III alone to Sf9 cells, and structural analysis shows that domain II and domain III have close interaction, suggesting that the presence of domain II is also important for cell binding.

### 2.4. Domains IV and V Are Glycan Binding Motifs 

**Figure 4:**
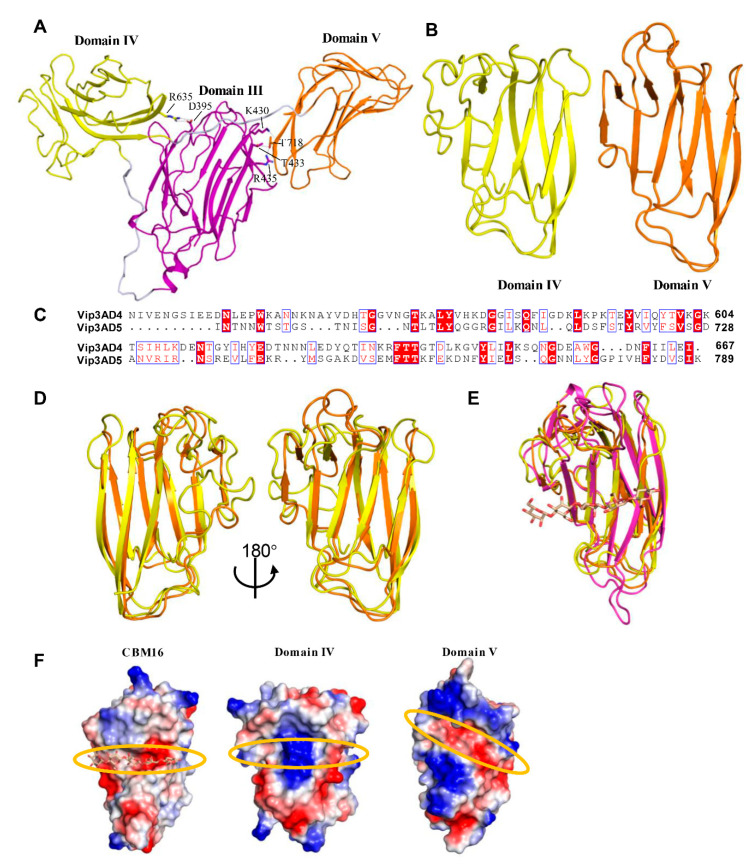
Domains IV and V of Vip3Aa11 have glycan binding motifs. (**A**) Domain architectures of domains III, IV and V of Vip3Aa11. The polar interactions between domain IV, V and domain III are shown as sticks. (**B**) Overall structure of Vip3Aa11 domain IV and V shown as a ribbon cartoon. (**C**) Amino acid sequence alignment between domain IV and domain V of Vip3Aa11. The identical residues are denoted in white characters and red background, and the similar residues are denoted in red. ClustalX2 was used to perform the sequence alignment [35]. ESPript-3.0 was used to generate the figure [36]. (**D**) Two views of structure superimposition between domain IV and domain V of Vip3Aa11 shown as a ribbon cartoon. Color of each domain is consistent with Figure 4B. (**E**) Structure superimposition between domains IV, V of Vip3Aa11 and glycan bound CBM16 (RCSB ID 2ZEY) shown as a ribbon cartoon. Domains IV and V are colored as Figure 4B, and CBM16 is shown in magenta color. The glycan in CBM16 is shown as stick in light brown color. (**F**) Surface charge distribution of the sugar-binding pocket of CBM16 (RCSB ID 2ZEY) and potential sugar-binding pocket of domains IV, V of Vip3Aa11, highlighted with the orange circle.

Both domains IV and V are all β-sheets folds (Figure 4A,B). Unlike domains II and III, which have compact organization, domains III/IV and IV/V are connected by long and flexible loops (Figure 4A). In addition to these loops, there are several polar interactions between domains IV/V and domain III, that reduce the flexibility and fix domains IV and V at the observed positions and orientations (Figure 4A). 

Domains IV and V are both built from two anti-parallel sheets of β sandwich, forming the “jelly-roll” topology. Despite showing only 17% sequence identity (Figure 4C), domains IV and V align very well structurally, with a root-mean-squared deviation (r.m.s.d) of 1.299 Å over 61 Cα atoms (Figure 4D). To examine the potential function of these two domains, we searched for their structural homologues using the DALI server [32]. The results for both domains show a very high similarity (Z score > 10) to family 16 carbohydrate binding module (CBM16) of S-Layer associated multidomain endoglucanase (RCSB ID 2ZEY). Superimposition of domains IV, V and CBM16 demonstrates that these three motifs share a similar fold (Figure 4E), suggesting that they are likely to share a related function as well. CBM16 is a carbohydrate-binding domain of the highly active mannanase from the thermophile *Thermoanaerobacterium polysaccharolyticum* with high specificity toward β-1,4-glucose or β-1,4-mannose polymers [34]. Analysis of the electrostatic surface potential shows that both domains IV and V have a surface pocket at a similar position to a sugar-binding pocket of the CBM16 domain (RCSB ID 2ZEY), although all three pockets have different shapes and charge distributions (Figure 4F). Taken together, our structural analysis indicates that domains IV and V of Vip3A both contain a conserved glycan binding motif and that these motifs may target different sugars.

### 2.5. Purified Vip3Aa11_200–end_ Has no Insecticidal Activity 

**Figure 5:**
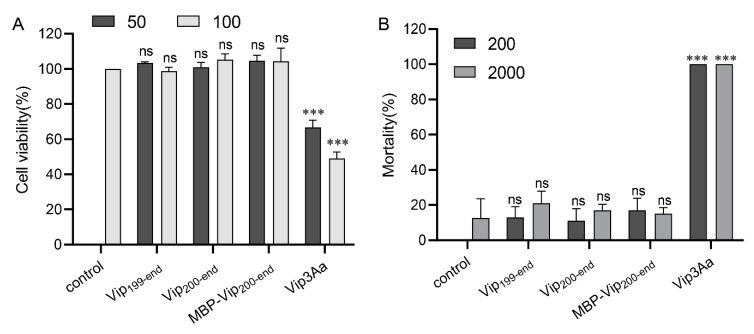
Cytotoxicity assays and insect bioassays of different Vip3A constructs. (**A**) Cell viability of Sf9 treated with different Vip3A constructs (50 and 100 μg/mL). (**B**) Mortality analysis of *S. exigua* caused by different Vip3A constructs (200 and 2000 ng/cm^2^). Data are expressed as the mean ± SD from three independent experiments; ns, nonsignificant; *** *p* < 0.001; one-way ANOVA with Dunnett’s method.

The C-terminal fragment of Vip3A has been considered to be the toxic core [8]. To verify whether the purified Vip3Aa_200–end_ still have insecticidal activity, cytotoxicity assays and insect bioassays were carried out. As shown in Figure 5A, the purified full length Vip3Aa toxin has significant toxicity to Sf9 cells, while Vip3Aa_199–end_, Vip3Aa_200–end_ and MBP-Vip3Aa_200–end_ have no toxicity to Sf9 cells. In addition, bioassay results showed that wild-type Vip3Aa was highly toxic against *S. exigua* at the concentration of 200 ng/cm^2^. However, the purified Vip3Aa_199–end_, Vip3Aa_200–end_ and MBP-Vip3Aa_200–end_ have no obvious insecticidal activity to *S. exigua* larvae even at the concentration of 2000 ng/cm^2^ (Figure 5B). These results indicate that the purified C-terminal fragment of Vip3A alone has no insecticidal activity.

## 3. Discussion

Vip3A toxins show a wide spectrum of specific insecticidal activities and are functionally distinct compared to the Cry toxins. These features make them good candidates for combined application with Cry toxins in transgenic crops to broaden the insecticidal spectrum and to prevent or delay resistance [1,8,11]. The structural features and insecticidal mechanisms of Cry toxins have been studied in detail, which has been crucial to their widespread application [2,3,4,6]. However, despite the fact that Vip3A toxins were identified almost 25 years ago [24], their mode of action remains poorly understood. One of the main reasons for this is the lack of a high-resolution three-dimensional structure, which significantly impedes detailed molecular-level functional and mechanistic studies, and thus limits the development of their insecticidal potential. In this study, we report the first crystal structure of the C-terminal fragment of Vip3A toxin, which provides a badly-needed framework to explore the molecular-level functional details of Vip3A-family toxins. 

Although the amino acid sequence similarity between the Vip3A family toxin and the 3d-Cry toxin is very low, our three-dimensional structural analysis showed interesting convergent evolution between these two families. Domain II of Vip3A has an all α-helix fold, including two conserved hydrophobic α-helices. Similarly, domain I of 3d-Cry also has an all α-helix fold and two hydrophobic α-helices, although it has additional α helices surrounding the conserved hydrophobic helices [4,37]. Several studies have reported that domain I of 3d-Cry toxin is involved in its membrane insertion and pore formation processes through its conserved hydrophobic α-helices [4]. This therefore suggests that domain II of Vip3A may also take part in these processes through its conserved hydrophobic α-helices. 

Both domain III of Vip3A and domain II of 3d-Cry are comprised of three β sheets with a conserved hydrophobic core. Extensive studies of domain II of 3d-Cry toxins showed that it plays a key role in the recognition of midgut receptors [4]. The results of our cell binding assay indicate that Vip3A domain III is also central to cell binding. Furthermore, the binding ability of domains II-III to Sf9 cells is similar to that of full-length Vip3Aa and stronger than domain III alone, and the Vip3Aa11_200–end_ structure shows that domain II and domain III have very compact interaction, which revealed that domain II is also involved in the binding of Vip3A to sensitive cells.

Domain III of 3d-Cry toxins was predicted to bind glycans with a classic glycan binding motif [38,39,40]. Based on amino acid sequence analysis, previous studies also predicted that all Vip3A proteins contain a carbohydrate-binding motif (CBM_4_9 superfamily; pfam02018) in the C-terminus (amino acids 536 to 652 in Vip3Aa) [8]. In the present structure, we found that, instead of the single CBM found in Cry toxins, there were two potential different CBM domains in the C-terminus of the Vip3A toxin, forming domains IV and V, respectively. Our structural analysis indicates that the putative glycan-binding pockets of these two domains differ significantly, suggesting that they are likely to have different glycan binding specificities. This multiplicity of CBMs in Vip3A toxins may increase the diversity of their target polysaccharides. However, in our cell binding assay, domains IV-V did not show binding ability to the Sf9 cells (Figure 3D,E), which may be due to the lack of the specific glycans recognized by domain IV-V on the Sf9 cells’ surface. The effect of domains IV and V on the toxicity of Vip3A toxins in insect midgut needs further study.

Taken together, we find here that although the overall structure and domain organization are very different between Vip3 toxin and 3d-Cry toxin, these two families are comprised of functionally and structurally related modules that are assembled in different ways, which may expand the insecticidal spectrum of Bt and make Bt more powerful and efficient to target and kill its hosts.

In addition, the ~65 kDa C-terminal fragment of Vip3A used to be considered as the toxic core [8]. However, recent studies indicated that the ~20 kDa N-terminal fragment and the ~65 kDa C-terminal fragment of Vip3A still bind together after digestion, and the N-terminus is required for the stability and toxicity of Vip3A [20,21,41]. Moreover, several studies further demonstrated that Vip3A remains tetrameric after being activated by trypsin or midgut fluid [20,22]. In our work, the C-terminal fragment of Vip3Aa alone has shown no toxicity through cytotoxicity assays and insect bioassays, and it forms a dimer in the crystal structure, which is consistent with the fact that the C-terminal fragment of Vip3B2160 will form a dimer instead of a tetramer without the N-terminal 21-kDa segment [30]. It is possible that, without N-terminal assistance, the C-terminal fragment cannot correctly assemble into an active tetramer; or, maybe without the protection of N-terminal, the C-terminal fragment loses stability and is degraded by protease.

**Figure 6:**
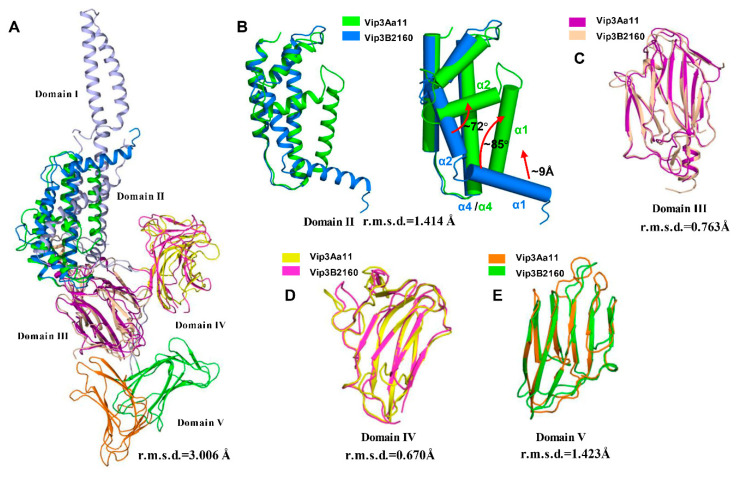
Structural comparation between corresponding domains of Vip3Aa11_200–end_ and Vip3B2160. (**A**) Structural comparation of Vip3Aa11_200–end_ and Vip3B2160. The domain I of Vip3B2160 which is lacking in Vip3Aa11_200–end_ structure is colored in light purple. (**B**) Structural overlay of domain II between Vip3A (green) and Vip3B2160 (blue). The cylindrical cartoon shows a detailed view of the conformational changes. (**C**–**E**) Structure superimposition for domain III, domain IV and domain V between Vip3Aa11_200–end_ and Vip3B2160. Each domain is color-coded as the indication.

Vip3A and Vip3B share about 65% sequence similarity and have different insecticidal specificity [42], and recently the C-terminal fragment was found to be related to insecticidal specificity of Vip3 [42,43]. Our structure provides a good opportunity to further study the mechanism of insecticidal specificity between Vip3A and Vip3B. The recently reported structure of Vip3B2160 showed a five-domain organization [30] (Figure S6). When these two Vip3 protein structures are superposed, the C-terminal fragment of Vip3B2160 shows similar folds and organization to Vip3Aa11 (Figure 6A). According to our division, the domain I of Vip3B2160, which is lacked in Vip3Aa11_200–end_ structure, formed a unique fold containing five α-helices wrapping around domain II. Domains III, IV and V of Vip3B2160 have similar folds as their counterparts from Vip3Aa11, respectively (Figure 6C–E). Although domain V in the two structures share similar folds, their positions in their respective structures are obviously different, which suggests that the location of domain V is flexible, and this flexibility of domain V may be related to the insecticidal specificity of Vip3 toxins. However, there are dramatic conformational differences in their domain II (Figure 6B); in the Vip3B2160 structure, the highly conserved hydrophobic α-helix (corresponding to the helix α4 in Vip3Aa11_200–end_ domain II) is surrounded by other helices from domains I and II (Figure 6A, Figure S6). In Vip3Aa11_200–end_ structure, the helices α1 and α2 of domain II have significant conformational changes and expose the hydrophobic region in domain II (Figure 6B). Hence, we hypothesize that the structural difference in domain II between the full-length and cleaved Vip3 proteins may represent the conformational change after the proteolysis of Vip3A toxins inside the insect midgut. In this scenario, once the cleavage site between domain I and II is processed by insect midgut juice, the α-helices of domain II may undergo a dramatic structural shift that enables helix α1 to rotate and form a hairpin-like structure with helix α4. However, a complex structure of ~20 kDa N-terminal fragment and the ~65 kDa C-terminal fragment of Vip3 after protease digestion will be needed to further prove this hypothesis and to further understand the function of the N-terminal fragment for Vip3 insecticidal activity.

Collectively, these data provide important structural and functional insights into Vip3A family toxins as well as a valuable resource to guide future studies and to re-evaluate the previous genetic and functional studies that are crucial for the development of Vip3A as a new generation of bio-insecticides.

## 4. Materials and Methods 

### 4.1. Bacterial Strains, Cell Lines and Plasmids

*E. coli* BL21(DE3) for plasmid constructions and protein purification were cultured at 37 °C in lysogeny broth (LB) or agar. Methionine auxotrophic *E. coli* strain B834 (DE3) (Novagen, Madison, WI, USA) were used for selenomethionine-substituted (SeMet) Vip3Aa_200–end_ expressing. The S. frugiperda Sf9 cells (Thermo fisher Scientific, Grand Island, NY, USA) were maintained and propagated in Sf-900 II SFM (Gibco, Grand Island, NY, USA) culture medium at 27 °C.

The DNA of Vip3Aa_200–end_ was amplified from the Vip3Aa11 gene (GenBank accession No. AY489126.1) using oligonucleotide primer Vip200-F and Vip200-R and cloned into the pET28a vector with an N-terminal 6×His-MBP tag. Plasmids used for RFP (red fluorescent protein) and C-terminal RFP tagged Vip3Aa (Vip3Aa-RFP) expression were constructed as described by Jiang et al. [11]. The different Vip3Aa DNA truncations were amplified from the Vip3Aa11 gene using oligonucleotide primer pairs, DmI-III-F and DmI-III-R, DmIV-V-F and DmIV-V-R, DmI-II-F and DmI-II-R, DmII-III-F and DmII-III-R, and DmIII-F and DmIII-R, and cloned into the pET28a vector with a C-terminal RFP-6×His tag, respectively. All plasmids were generated by the Gibson assembly strategy [44]. The nucleotide sequences of recombinant plasmid were verified by DNA sequencing. All the primers used in this study are shown in Table S2.

### 4.2. Protein Expression and Purification

Native His-MBP-Vip3Aa_200–end_ (Vip3Aa_200–end_) protein was expressed in *E. coli* B21(DE3) at 25 °C for 48 h in autoinduction Terrific broth (TB) medium. The cells were harvested by centrifugation at 5000× *g* at 4 °C for 15 min and the pellet was resuspended in lysis buffer (20 mM Tris–HCl pH 8 and 150 mM NaCl). After the cells were lysed by high pressure cell crusher (Union-Biotech co., LTD, shanghai, China), the supernatant was collected after centrifuged at 12000× *g* at 4 °C for 60 min. The proteins were purified using Ni-NTA agarose resin, washed with 20 mM Tris-HCl, 150 mM NaCl, 20 mM imidazole, pH 8.0, and then eluted with 300 mM imidazole. The Vip3Aa_200–end_ proteins were further purified by HiTrap Q HP ion-exchange chromatography and Superdex 200 gel filtration chromatography (GE Healthcare Life Sciences, Marlborough, MA, USA). Fractions containing the Vip3Aa_200–end_ protein were concentrated to ~7 mg/mL for crystallization. The expression and purification steps of other Vip3Aa truncations were the same as those of Vip3Aa_200–end_.

SeMet-substituted Vip3Aa_200–end_ was expressed in *E. coli* B834(DE3) strain. Briefly, the cells were cultured in the LB medium at 37 °C along with shaking until the OD600 of the bacterial culture reached 1.0. The cells were harvested by centrifugation at 4000× *g* at 4 °C for 15 min and the pellet was washed once with PBS. The pellet was resuspended in 1 L Medium A (M9 medium plus 0.4% glucose, 1 mM MgCl_2_, 1 mM CaCl_2_, 1 mg Biotin, 1 mg thiamin, 50 mg EDTA, 8.3 mg FeCl_3_, 0.84 mg ZnCl_2_, 0.13 mg CuCl_2_, 0.1 mg CoCl_2_, 0.1 mg H_3_BO_3_ and 0.016 mg MgCl_2_) and incubated for 3 h at 37 °C. We added 50 mg seleno-methionine in the medium and incubated for a further 30 min. The protein was incubated to express for a further 10 h by adding 200 mM IPTG (isopropyl-β-D-thiogalactopyranoside). The SeMet-Vip3Aa_200–end_ was purified by the same procedure as for the native Vip3Aa_200–end_ protein.

### 4.3. Crystallization, Data Collection and Structural Determination 

The purification of His_6_-tagged MBP-Vip3Aa_200–end_ used for crystallization is described above. MBP-Vip3Aa_200–end_ (5 mg/mL) was used to perform initial spare matrix crystal screening with a crystallization robot. After crystal optimization trials, MBP-Vip3Aa_200–end_ (7 mg/mL) crystals grew in 3 days at 18 °C using the hanging-drop vapor-diffusion method in a mix of 1 μL of protein with 1 μL of reservoir solution consisting of 0.1 M sodium acetate pH 4.2, 0.5 M potassium formate, 0.1 M ammonium sulfate and 11% PEG4000. SeMet MBP-Vip3Aa_200–end_ crystals grew in a similar condition. 

A native data set with the space group of P2_1_2_1_2_1_ was collected at 3.62 Å (native I). A weak selenomethionine (SeMet) derivative data set was collected at 3.9 Å with the same symmetry as the native I crystal for the amino-acid assignment using the difference Fourier map of the SeMet derivative. After further crystallization optimization, another native crystal (native II) was obtained with the space group of P2_1_ that could diffract to around 3.2 Å. Diffraction data were collected on BL17U1 and BL18U beamlines at Shanghai Synchrotron Radiation Facility (Shanghai, China) and processed by HKL2000 [45].

Molecular replacement was carried out to identity the MBP positions in the native crystals [46] by PHASER [47]. The initial phases were further improved with multi-crystal averaging [48]. Model building was performed manually in COOT [49], and the sequence assignment was helped with the SeMet anomalous difference map. Figures were prepared using PyMol (v.2.3.2, https://pymol.org/, Schrödinger, New York, NY, USA). Structure refinement was done by PHENIX [50]. The data collection and refinement statistics are summarized in Table S1.

### 4.4. Immunofluorescence

Sf9 cells with a density of 5 × 10^4^ cells per ml were seeded into 6-well culture plates separately. After overnight culture, the cells were respectively treated with RFP tagged Vip3Aa or its truncations (0.15 μM) for 6 h. After treatment, the cells were washed three times with PBS to remove unbound proteins, and fixed with 4% paraformaldehyde at 37 °C for 15 min. The cell nuclei were labeled with DAPI (0.2 μg/mL) for 30 min. Cell images were captured using a Nikon TI-E inverted fluorescence Microscope (Nikon, NIKON TI-E, Tokyo Metropolis, Japan).

### 4.5. Cytotoxicity Assays

Cell viability assays were performed as described by Jiang et al. [25]. Briefly, cells with a density of 5 × 10^4^ cells per ml were seeded into 96-well culture plates separately. After overnight incubation, the cells were treated with different Vip3Aa toxins for 72 h. WST-8 reagent was then added to each well. After incubating at 27 °C for 2 h, the absorbance was measured in microplate reader at 450 nm. Treatment with protein buffer was used as a control. All tests were performed in triplicate and were repeated at least three times. Cell viability (%) = average absorbance of treated group/average absorbance of control group × 100%.

### 4.6. Bioassay

Bioassays were assessed using surface contamination method with *S. exigua* first instar larvae and maintained in a rearing chamber at 27 °C, with 50% relative humidity, and 16:8 h light:dark photoperiod. The artificial diet was poured in a 1.8-cm^2^ 24 well plate (about 5 mm thick per hole). 200 and 2000 ng/cm^2^ concentrations of Vip3Aa proteins (full length Vip3Aa, Vip3Aa_199–end_ Vip3Aa_200–end_ and MBP-Vip3Aa_200–end_) were spread on the diet. A tris buffer (20 mM Tris-HCl, 300mM NaCl, pH 8.0) was used as a blank control. Three independent replicates and 16 first instar larvae of *S. exigua* were used for each concentration. Mortality was recorded after 5 days. 

### 4.7. Statistical Analysis

All functional assays were performed at least three times independently. Data were shown as means ± SD. All statistical data were calculated using GraphPad Prism version 8.0 (GraphPad Software, San Diego, CA, USA). One-way ANOVA followed by Dunnett’s test were used to identify statistically significant differences between treatments. Significance of mean comparison is annotated as follow: ns, nonsignificant; **, *p* < 0.01; ***, *p* < 0.001. A *p* value of < 0.05 was considered to be statistically significant.

### 4.8. Data Availability

Coordinate for the atomic structure has been deposited in the RCSB Protein Data Bank under RCSB ID: 6VLS. The data that support the findings of this study are available from the corresponding author upon reasonable request.

## Supplementary Materials

The following are available online at https://www.mdpi.com/2072-6651/12/7/438/s1, Figure S1. Sequence alignment of selected Vip3 family members. Each domain is indicated by the lines bellow sequences, colored as in Figure 1A. Secondary structural elements of Vip3Aa11 are shown above the sequences. The conserved hydrophobic amino acid residues discussed in domain II and domain III are marked with green and magenta triangles, respectively. The potential cleavage site between domain III and domain IV is highlighted with blue triangle. ClustalX2 was used for the sequence alignment. ESPript-3.0 was used to generate the figure. Figure S2. Structure of MBP-Vip3Aa11_200–end_ in the *P*2_1_ space group. Two views of MBP-Vip3Aa11_200–end_ structure in one asymmetric unit. There are four molecules of MBP-Vip3Aa11_200–end_ in one asymmetric unit and they are arranged into two copies of dimer in the different orientations. The molecule A, B, C and D are shown in green, cyan, magenta and yellow, respectively. The MBP (Maltose Bind Protein) tags are shown in silver color in all four molecules. The interaction area between molecule B and C is less than 500 Å^2^, as calculated by PISA server. Figure S3. Structural alignment between Molecule A and B from the Vip3Aa11_200–end_ dimer. Structure superimposition for the Vip3Aa11_200–end_ and each domain between molecule A and B from the Vip3Aa11_200–end_ dimer structure. Molecule A is colored as Figure 1A, and Molecule B is shown in cyan color. The root means square deviation (r.m.s.d) of each alignment is listed. Figure S4. Two hydrophobic helices from domain II. The hydrophobic amino acid residues are shown as sticks and labelled with residue numbers. The amino acid residues involved in the hydrophobic (red) and polar (yellow) interactions between α1 and α4 helices are shown as sticks, and the polar interactions are shown in black dashes. Figure S5. Images of Sf9 cells treated with RFP. Fluorescence microscope images of Sf9 cells treated with RFP protein only for 6 h as control. The images are representative of three independent experiments. Nuclei are stained with DAPI (blue), Scale bar, 10 μm. Figure S6. Overall structure of Vip3B2160. Domains I, II, III, IV and V are colored in light purple, blue, light brown, magenta and green, respectively. Table S1. X-ray and refinement statistics. Table S2: Primers used in this study
