# Supplementary material for: Structural and Functional Insights into the C-terminal Fragment of Insecticidal Vip3A Toxin of Bacillus thuringiensis
Source: Toxins (Basel). 2020 Jul 5;12(7):438. doi: 10.3390/toxins12070438 (PMC7404976; doi:10.3390/toxins12070438)
Supplement: Supplementary file 1 [file toxins-12-00438-s001.pdf]

Supplementary Materials: Structural and Functional Insights into the C-terminal Fragment of Insecticidal Vip3A Toxin of *Bacillus thuringiensis*

Kun Jiang, Yan Zhang, Zhe Chen, Dalei Wu, Jun Cai and Xiang Gao

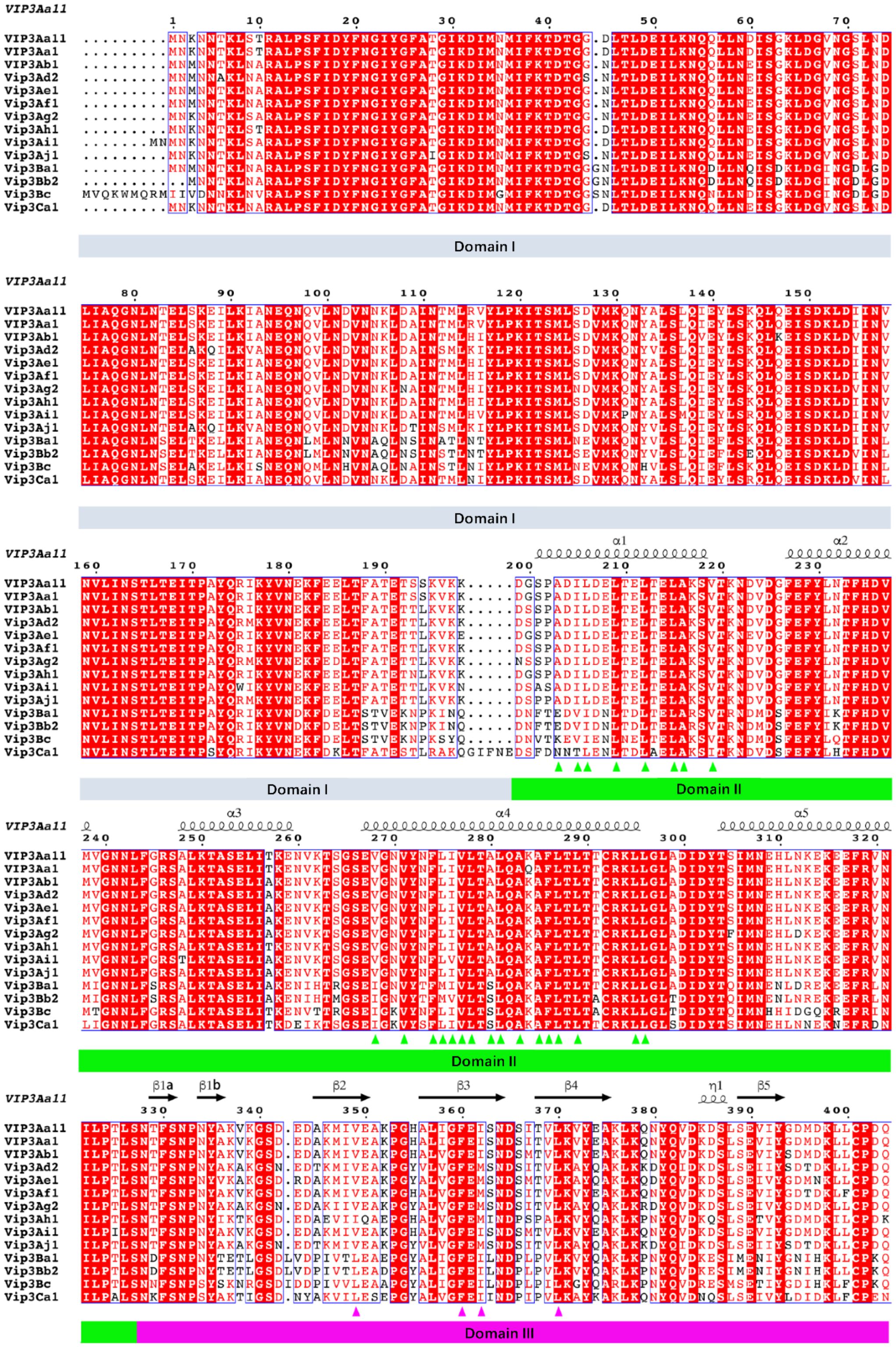

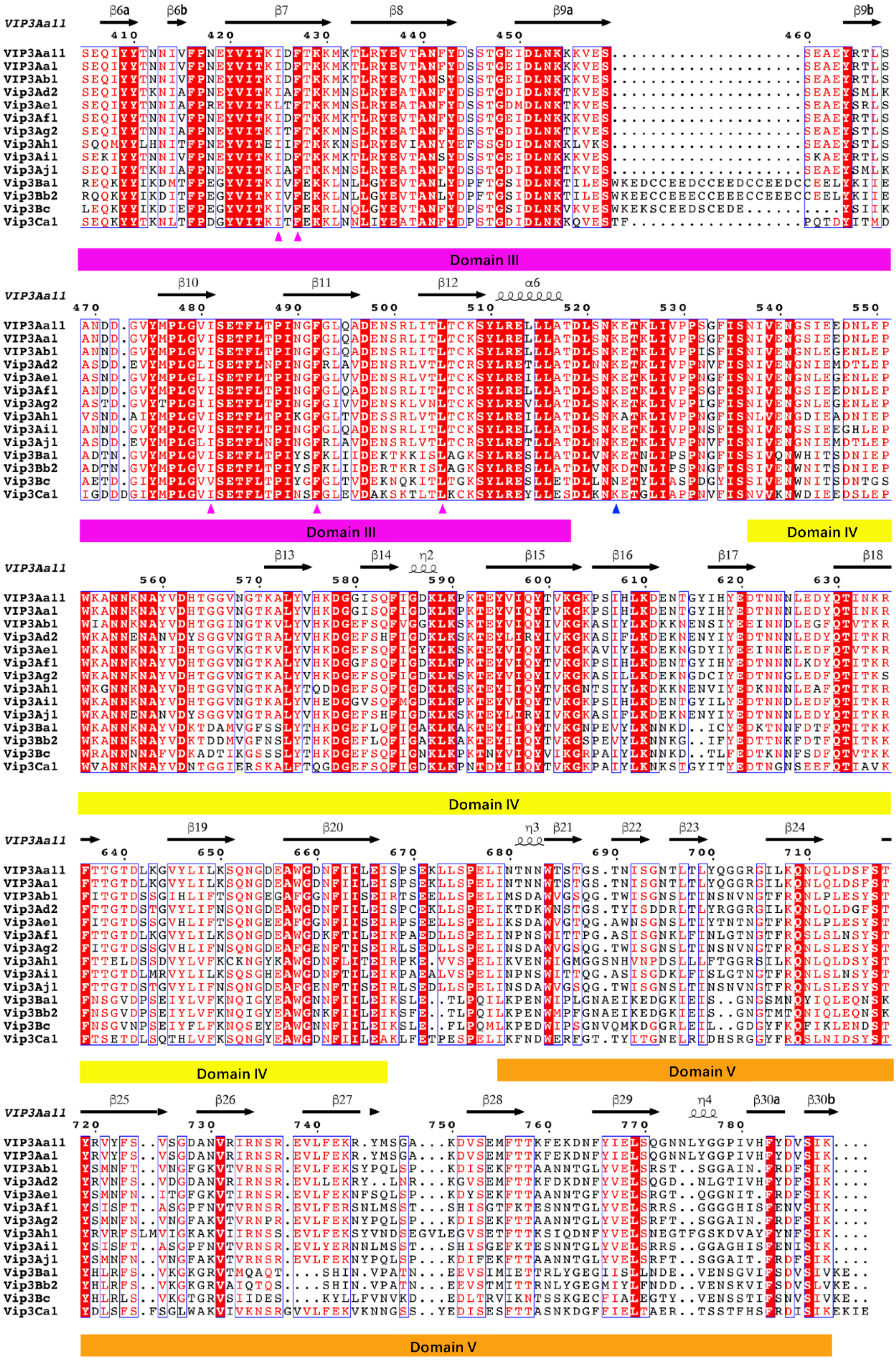

Figure S1. Sequence alignment of selected Vip3 family members. Each domain is indicated by the lines below the sequences, coloured as in Figure 1A. Secondary structural elements of Vip3Aa11 are shown above the sequences. The conserved hydrophobic amino acid residues discussed in domain II and domain III are marked with green and magenta triangles, respectively. The potential cleavage

site between domain III and domain IV is highlighted with blue triangle. ClustalX2 was used for the sequence alignment. ESPript-3.0 was used to generate the figure.

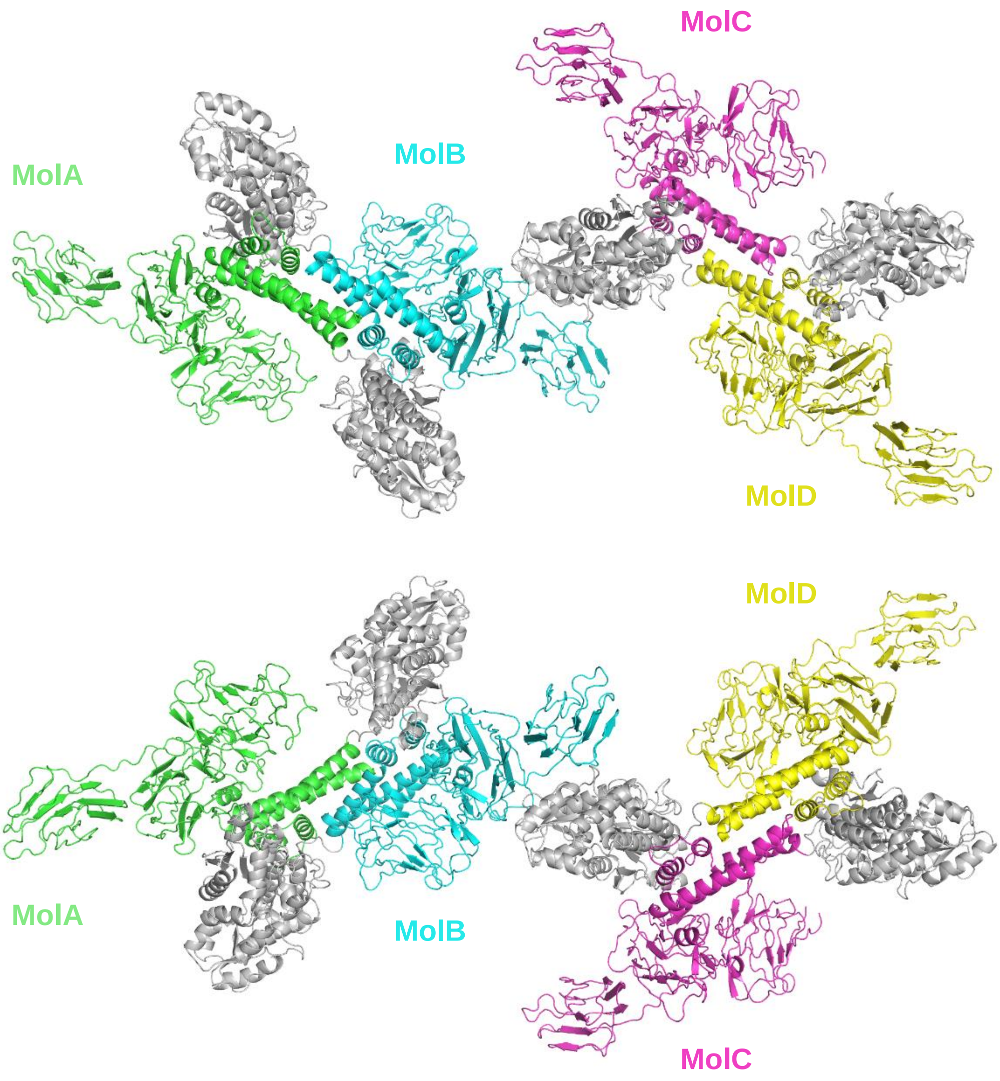

Figure S2. Structure of MBP-Vip3Aa11$_{200\text{–}end}$ in the *P*2$_1$ space group. Two views of MBP-Vip3Aa11$_{200\text{–}end}$ structure in one asymmetric unit. There are four molecules of MBP-Vip3Aa11$_{200\text{–}end}$ in one asymmetric unit and they are arranged into two copies of dimer in the different orientations. The molecule A, B, C and D are shown in green, cyan, magenta and yellow, respectively. The MBP (Maltose Bind Protein) tags are shown in silver color in all four molecules. The interaction area between molecule B and C is less than 500 Å^2, as calculated by PISA server.

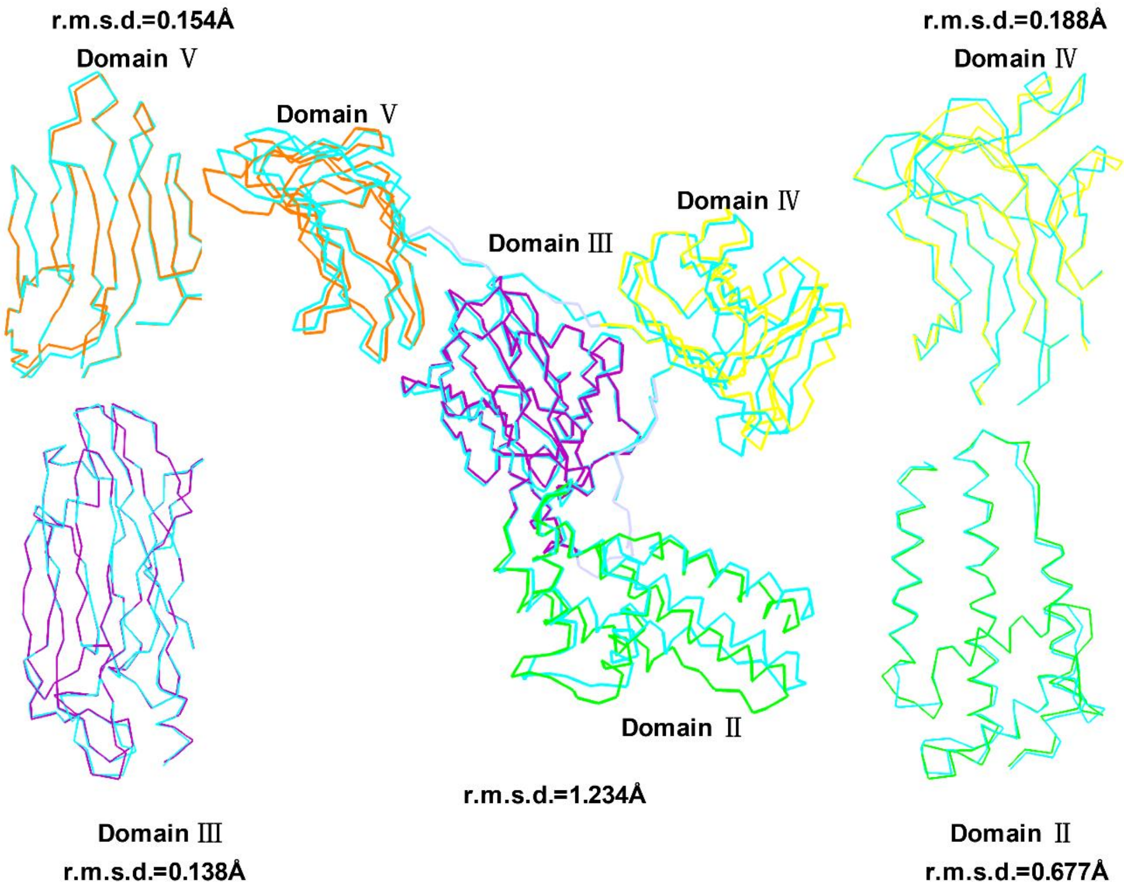

Figure S3. Structural alignment between Molecule A and B from the Vip3Aa11200–end dimer. Structure superimposition for the Vip3Aa11200–end and each domain between molecule A and B from the Vip3Aa11200–end dimer structure. Molecule A is coloured as Figure 1A, and Molecule B is shown in cyan color. The root mean square deviation (r.m.s.d) of each alignment is listed.

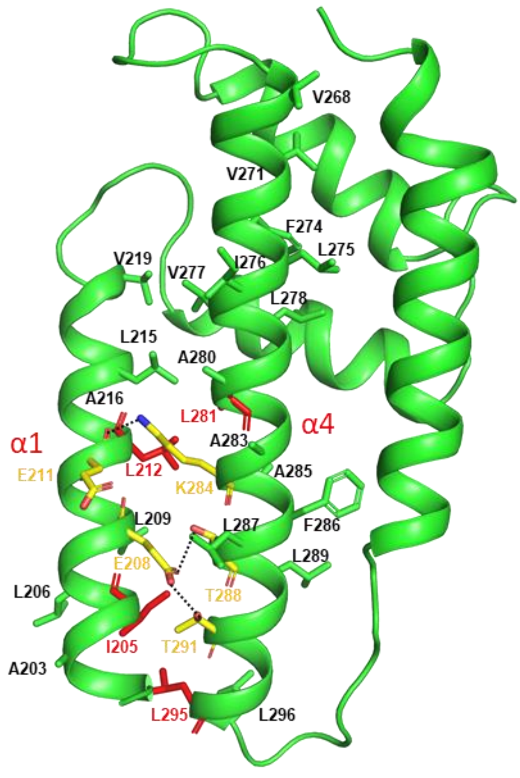

Figure S4. Two hydrophobic helices from domain II. The hydrophobic amino acid residues are shown as stick and labelled with residue numbers. The amino acid residues involved in the hydrophobic (red) and polar (yellow) interactions between α1 and α4 helices are shown as sticks, and the polar interactions are shown in black dashes.

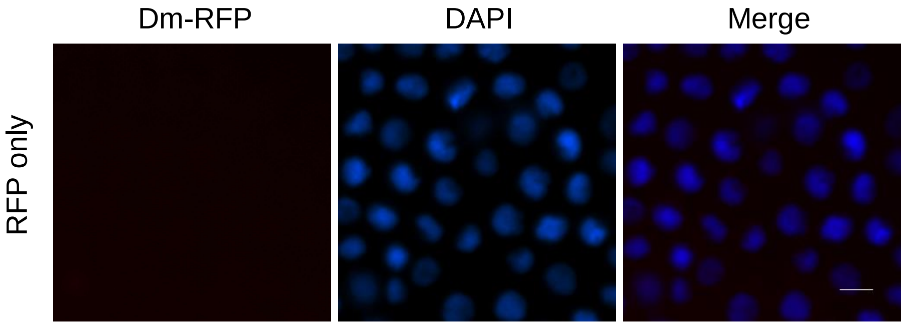

Figure S5. Images of Sf9 cells treated with RFP. Fluorescence microscope images of Sf9 cells treated with RFP protein only for 6 h as control. The images are representative of three independent experiments. Nuclei are stained with DAPI (blue), Scale bar: 10 μm.

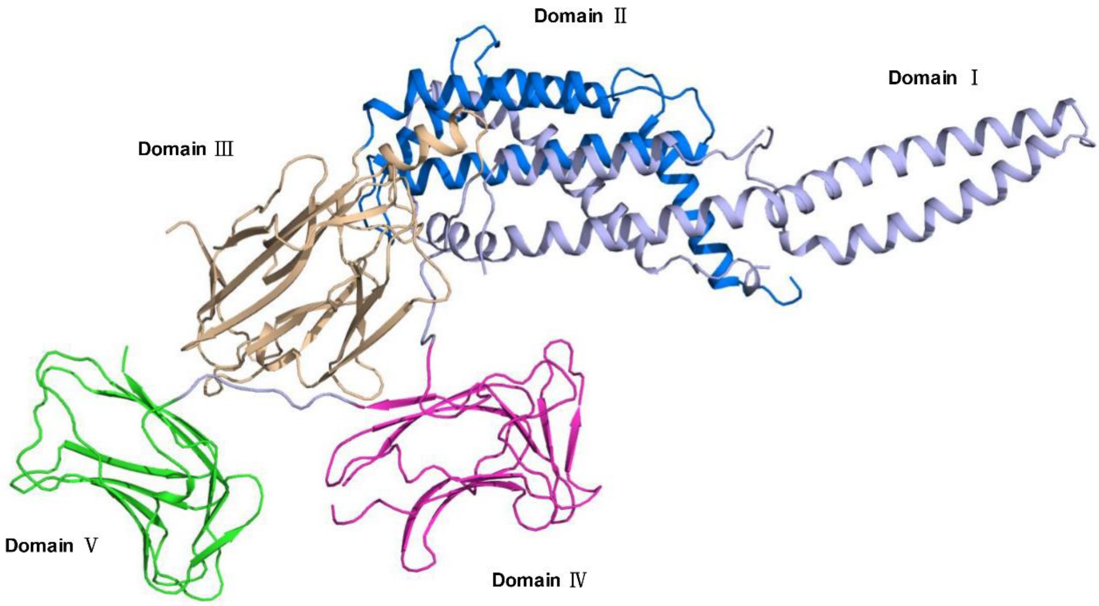

Figure S6. Overall structure of Vip3B2160. Domains I, II, III, IV and V are coloured in light purple, blue, light brown, magenta and green, respectively.

Table S1. X-ray and refinement statistics.

**Parameter name**	**Se-SAD**	**Native I**	**Native II**
Data collection			
Wavelength (Å)	0.9792	0.9793	0.9793
Space group	P212121	P212121	P21
Cell dimensions			
a, *b*, *c* (Å)	125.71,139.35,163.20	126.08,140.20,167.64	136.09,127.88,149.18
α, β, γ (°)	90, 90, 90	90, 90, 90	90,91.378,90
R_{meas}	0.153 (0.829)*	0.207(0.915)	0.145 (0.753)
$I/\sigma I$	9.30(2.80)	7.47(1.32)	6.11 (1.00)
CC1/2	0.981(0.674)	0.983(0.565)	0.993(0.541)
Completeness (%)	91.25 (79.60)	93.28(91.40)	97.33 (95.63)
Redundancy	3.5 (3.7)	3.9(3.8)	2.3 (2.3)
Wilson B-factor	48.4	99.2	71.4
MR-SAD Phasing			
Selenium sites (ShelX/D)	11		
PATFOM (/D)	24.47		
Overall CC (ShelX/E) (%)	6.57		
Pseudo-Free CC (/E) (%)	60.29		
Final Map CC (/E)	0.995(0.897)		
Figure of Merit (FOM)	0.564		
Refinement			
Resolution (Å)	49.93–3.91 (4.04–3.90)	42.42–3.62 (3.75–3.62)	28.93–3.195 (3.309–3.195)
No. reflections			82266 (8040)
R_{work}/R_{free}			0.1980/0.2389
No. atoms			30179
Protein			29960
Ligand/ion			42
Water			177
Average *B*-factors			74.14
Protein			74.32
Ligand			86.70
Water			40.48
R.m.s. deviations			
Bond lengths (Å)			0.002
Bond angles (°)			0.58
Ramachandran			
Favored (%)			95.01
Allowed (%)			4.80
Outliers (%)			0.18

*Values in parentheses are for highest-resolution shell.\

Table S2. Primers used in this study.

Primer Name	Sequence(5′→3′)	Function
Vip200-F	GAGCTTTCGCTGCAGCGTCCGGCTCTCCTGCAGATATTC	$Vip_{200–end}$ cloning
Vip200-R	GTTAGCAGCCGGATCTCAGTGTTACTTAATAGAGACATCGTAAAAATGTAC	$Vip_{200–end}$ cloning
DmI-III-F	AAGAAGGAGATATACCATGGGCATGAACAAGAATAATACTAAATTAAGC	DmI-III cloning
DmI-III-R	TCGACTGCAGAGGCCTGCATAGAAAGTGTAGGGAGGATGTTTAC	DmI-III cloning
DmVI-V-F	AAGAAGGAGATATACCATGGGCGGTTTTATTAGCAATATTGTAGAG	DmVI-V cloning
DmVI-V-R	TCGACTGCAGAGGCCTGCATCTTAATAGAGACATCGTAAAAATG	DmVI-V cloning
DmI-II-F	AAGAAGGAGATATACCATGGGCATGAACAAGAATAATACTAAATTAAGC	DmI-II cloning
DmI-II-R	TCGACTGCAGAGGCCTGCATAGAAAGTGTAGGGAGGATGTTTAC	DmI-II cloning
DmII-III-F	AAGAAGGAGATATACCATGGGCGATGGCTCTCCTGCAGATATTCTTG	DmII-III cloning
DmII-III-R	TCGACTGCAGAGGCCTGCATAGAAAGTGTAGGGAGGATGTTTAC	DmII-III cloning
DmIII-F	AAGAAGGAGATATACCATGGGCACACTTTCTAATACTTTTTCTAATC	DmIII cloning
DmIII-R	TCGACTGCAGAGGCCTGCATTTCTTTATTGCTTAAGTCTGTTGC	DmIII cloning
pET-MBP-F	CACTGAGATCCGGCTGCTAAC	pET28-MBP cloning
pET-MBP-R	TTCTTTATTGCTTAAGTCTG	pET28-MBP cloning
pET-RFP-F	ATGCAGGCCTCTGCAGTCGACGGG	pET28-RFP cloning
pET-RFP-R	GCCCATGGTATATCTCCTTCTT	pET28-RFP cloning

The nucleic acid bases corresponding to each gene are labeled with underscores.
